# Non-Contact, Mechanical Fatigue-Related ACL Injury Prevention Through Extracellular Matrix Crosslink Preservation: A Narrative Review

**DOI:** 10.3390/jfmk11020180

**Published:** 2026-04-29

**Authors:** John Nyland, Maggie Head, Essa H. Gul, Brandon Pyle, Jarod Richards

**Affiliations:** 1 Division of Sports Medicine, Department of Orthopaedic Surgery, University of Louisville, Louisville, KY 40202, USA; 2 School of Medicine, University of Louisville, Louisville, KY 40202, USA

**Keywords:** adolescent, anterior cruciate ligament, extracellular matrix, injury prevention, knee

## Abstract

**Background:** Anterior cruciate ligament (ACL) injuries are increasing in young athletes and many are related to non-contact, spontaneous mechanical fatigue-related ruptures. The objective of this narrative review is to identify and synthesize the anatomical, histological, physiological, and biomechanical basis of extracellular matrix (ECM) factors that contribute to ACL injuries and suggest ways to decrease their occurrence. **Methods:** The primary investigator searched PubMed, Web of Science, and Google Scholar database titles and abstracts using search phrases with Boolean operators: “anterior cruciate ligament” OR “ACL”, OR “cranial cruciate ligament” AND “disease”; “anterior cruciate ligament” OR “ACL”, OR “cranial cruciate ligament” AND “spontaneous rupture” OR “non-contact injury”; and “anterior cruciate ligament” OR ACL, OR cranial cruciate ligament” AND “crosslink”, “collagen” OR “extracellular matrix”; and “anterior cruciate ligament” OR “ACL”, OR “cranial cruciate ligament” AND “microtrauma”, OR “sudden” OR “fatigue failure”. The primary investigator and a sports orthopedic surgeon reviewed titles and abstracts of diverse evidence sources. From these identified sources, the study team performed full text reviews, selected contributing articles, performed Strength of Recommendation Taxonomy (SORT) grading, and synthesized the following themes: A Hostile Environment, ACL Strain, and Poor Nutrient Delivery; Accumulative ACL Microtrauma and Mechanical Failure; The ACL Differs From Other Ligaments; Collagen, the ECM, and ACL Mechanobiology; Crimps and ACL ECM Stretch; Crosslinks Improve ECM Mechanical Properties; The Delicate Collagen Synthesis and Degradation Balance; Exercise Training and the ACL; Can Nutraceuticals Help Restore the Balance?; Training Induced ACL Hypoxia; Estrogen and the Female Athlete; Counting Pitches or Counting Collagen Fiber Ruptures; and Restoring A Positive Anabolic–Catabolic Collagen Balance. **Results:** Regular exercise training within a physiologically safe loading range is vital to ACL ECM health. However, low or moderate evidence suggested that poor blood supply, slow metabolism, and a hypoxic environment may unbalance anabolic and catabolic homeostasis. Active rest and recovery concepts that prevent youth baseball shoulder and elbow injuries may help prevent non-contact ACL injuries. **Conclusions:** More prescriptive active rest and recovery intervals and neuromuscular control training may restore the anabolic–catabolic balance that increases mature crosslink density and improves ACL ECM strength. Confirmatory studies are needed to better establish therapeutic intervention mode(s), timing, dosage, and frequency optimization.

## 1. Introduction

To witness a young athlete with no previous knee injury history suddenly rupture their anterior cruciate ligament (ACL) while performing a simple, low-intensity pre-game warm-up activity is unforgettable. One might wonder how a healthy ACL could spontaneously rupture under such low-intensity, low-velocity loads. Compared to healthy conditions, impaired ACL vascularity from even seemingly minor recurrent trauma may increase medial femoral condyle chondral lesion risk by 3 times [1].

Osteoarthritis (OA) affects over 500 million people globally contributing to significant disability and medical costs, and one of the most prevalent knee OA causes is an ACL tear [2,3]. Little is known about how much a healthy knee can tolerate biochemical knee joint changes following minor trauma or overuse, or how best to restore homeostasis [1,2]. The lifetime post-trauma OA risk ranges from 20% to >50%, and despite the evolution of both surgical and non-surgical interventions, OA risk post-trauma has not decreased over the past 25 years [3]. Mild non-contact ACL injuries or biochemical knee joint environment changes may not immediately display clinically apparent signs or symptoms but nevertheless can lead to post-traumatic OA, particularly with meniscal deficiency or impaired function [3,4]. An improved understanding of the cellular and molecular events that lead to post-traumatic knee OA present an opportunity to develop new primary prevention strategies [4]. For this to occur we need to better understand spontaneous non-contact ACL rupture etiology [5,6,7]. A growing body of evidence suggests that knee joint biochemical responses to initial mechanical injury plays a key role in tissue damage onset and progression [4].

Since the 1990s, ACL tears in young athletes have increased 400% [8]. More than 70% of ACL injuries are associated with non-contact mechanisms [9,10] involving aggressive quadriceps femoris muscle group activation, excessive knee joint loads, awkward single leg landing or pivoting directional change maneuver biomechanics, trunk and lower extremity neuromuscular control deficits, and the accumulative effects of repetitive submaximal ligament loading events [11,12]. Despite the rising incidence of youth ACL injury, the etiological factors contributing to spontaneous ruptures are poorly understood. Even under high physiological strain, healthy connective tissues seldom rupture acutely unless degenerative signs such as small collagen fibril size and greater type III collagen concentrations exist [13,14]. Understanding ACL material properties and structural behavior under repetitive, submaximal loads is crucial [15]. When subjected to rapid loads, such as during athletic cutting/pivoting maneuvers or single leg jump landings, the ACL predominantly displays elastic mechanical properties as it attempts to return to its original shape. Prolonged, excessive, or highly repetitive loads, however, may cause viscoelastic creep, where the ACL slowly deforms over time, leading to accumulated microtrauma and eventual failure [16].

Sudden ACL ruptures lead to a 5 times greater knee OA risk 10–20 years post-injury [17]. Although a single submaximal ACL loading session is unlikely to rupture a healthy ACL, highly repetitive, submaximal loading with limited active rest and recovery between loading events may result in sudden mechanical failure [11,12,18]. Even one “sub-critical” ACL injury without any clinical signs may progress to spontaneous mechanical fatigue failure [17] as microtrauma accumulation from repetitive submaximal loading may propagate molecular-level collagen unraveling [19,20]. The objective of this narrative review is to identify and synthesize the anatomical, histological, physiological, and biomechanical basis of extracellular matrix (ECM) factors that contribute to ACL injuries and suggest ways to decrease their occurrence.

## 2. Materials and Methods

The primary investigator searched PubMed, Web of Science, and Google Scholar database titles and abstracts using the following search phrases with Boolean operators: “anterior cruciate ligament” OR “ACL”, OR “cranial cruciate ligament” AND “disease”; “anterior cruciate ligament” OR “ACL”, OR “cranial cruciate ligament” AND “spontaneous rupture” OR “non-contact injury”; and “anterior cruciate ligament” OR ACL, OR cranial cruciate ligament” AND “crosslink”, “collagen” OR “extracellular matrix”; and “anterior cruciate ligament” OR “ACL”, OR “cranial cruciate ligament” AND “microtrauma”, OR “sudden” OR “fatigue failure”. Database searches were performed in November of 2025. No publication year or language restrictions were used. The primary investigator and a sports orthopedic surgeon reviewed the titles and abstracts of diverse evidence sources including higher-quality prospective patient-oriented studies, animal models, histological studies, simulation or mechanistic studies, cadaveric models, basic science and more disease-oriented studies, reviews, book chapters, conceptual papers, theoretical models and expert opinions. After reviewing all sources, they selected 103 unique sources to contribute to the review. From these sources all authors performed full text reviews, performed Strength of Recommendation Taxonomy (SORT) evidence quality grading [21], and developed the following review themes from synthesized information: A Hostile Environment, ACL Strain, and Poor Nutrient Delivery; Accumulative ACL Microtrauma and Mechanical Failure; The ACL Differs From Other Ligaments; Collagen, the ECM, and ACL Mechanobiology; Crimps and ACL ECM Stretch; Crosslinks Improve ECM Mechanical Properties; The Delicate Collagen Synthesis and Degradation Balance; Exercise Training and the ACL; Can Nutraceuticals Help Restore the Balance?; Training Induced ACL Hypoxia; Estrogen and the Female Athlete; Counting Pitches or Counting Collagen Fiber Ruptures; and Restoring A Positive Anabolic–Catabolic Collagen Balance.

## 3. Results and Discussion

The study search results are displayed in Table 1 which follows review themes of synthesized information. The Fleiss kappa score statistic was 0.73, *p* < 0.001 suggesting substantial reviewer SORT grade agreement [22]. When reviewers disagreed about the SORT evidence level grade, the primary investigator and sports orthopedic surgeon decided upon the final grade. Section SORT evidence level grades are documented at the end of each theme.

### 3.1. A Hostile Environment, ACL Strain, and Poor Nutrient Delivery

Complete ACL rupture also includes its thin synovial sheath exposing the torn ECM to synovial fluid, inflammatory cytokines created by the synovial membrane, hemorrhagic breakdown products, and proteolytic enzymes [23]. With no connecting tissue scaffold, high cytokine concentration, and synovial fluid contact, the completely ruptured ACL has poor natural healing capacity. In contrast, the accumulated microtrauma preceding spontaneous ACL rupture generally occurs in a seemingly healthy tissue, with no previous injury history other than minor trauma or overuse, lacking any clinical signs or symptoms [17]. Therefore, with early identification and sufficient active rest and recovery, the microtraumatized ACL should possess better natural healing capacity [16,17]. This, however, depends on ACL cellular properties, how they respond to vascularly delivered growth factors (i.e., transforming growth factor-beta (TGF-β), and how well homeostatic metabolism is regulated [24,25,26].

Muscles possess greater regenerative or natural reparative healing potential than ligaments, particularly the ACL [27]. As a non-contractile tissue that resists potentially injurious three-dimensional knee joint loading forces over prolonged time periods, the ACL primarily functions anaerobically with a low metabolic rate and poor nutrient delivery from its limited vascularity or slow diffusion. As an intracapsular/extrasynovial tissue positioned within the femoral intercondylar notch, it is subjected to repetitive tensile and compressive loading forces. Morphological traits such as an increased posterior tibial slope, a convex lateral tibial plateau, a narrow femoral intercondylar notch, genu valgus or recurvatum, and coxa varus may also increase ACL loading forces [28,29].

During highly repetitive intense sport movements, prolonged or excessive ACL loads may compromise vascular nutrient delivery and metabolic byproduct waste removal [2,29] leading to reactive oxygen species (ROS) accumulation that structurally damages the ACL ECM by increased collagen degradation, cellular apoptosis, and chronic inflammation. During high-intensity exercise, in association with sympathetic nervous system mediated vasoconstriction, blood flow gets shunted away from organs like the kidneys and digestive system toward working lower extremity muscles [30]. Local nitric oxide, adenosine, and prostaglandin vasodilator release (“functional sympatholysis”) in working muscles overrides general sympathetic vasoconstriction to deliver oxygen-rich blood. This “exercise hyperemia” process is crucial for delivering oxygen and nutrients to active muscles while also removing metabolic waste products. Although observed in muscles, it does not occur at the ACL. Under prolonged lower limb load increases during high regional muscle activation, the ACL likely functions under even greater anaerobic, more hypoxic conditions, increasing ROS accumulation and ischemic tissue damage risk [2,31,32].

Cellular mechanotransduction converts mechanical signals into biochemical responses that are essential for maintaining ACL ECM homeostasis and for initiating post-injury repair [33]. Young athletes who participate in a single cutting or pivoting sport year-round, however, may be more likely to possess an ACL ECM that is affected by overtraining and under-recovery [6,7,18,34]. This is particularly problematic for females due to high estrogen concentrations during the late follicular menstrual cycle phase. Factors contributing to overuse and under-recovery may include high training intensity, frequency, duration, total volume, year-round single-sport specialization, and increased environmental and societal stressors [6,7,35,36]. Morphological, physiological, developmental, environmental, societal, personal, and behavioral factors may also place young athletes at greater risk of spontaneous non-contact ACL injury risk from an accumulated microtrauma etiology [33]. In summary, poor vascularity, slow metabolism, and prolonged hypoxic function in a synovial joint environment likely reduces and delays ACL ECM capacity for microtrauma healing. The SORT evidence levels for this theme were 2 [18,29,33] and 3 [2,6,7,16,17,23,24,25,26,27,30,31,32,34,35,36].

### 3.2. Accumulative ACL Microtrauma and Mechanical Failure

Collagen strength depends on extracellular triple-helix polypeptide chains self-assembled into collagen fibrils that are stabilized by intra- and intermolecular enzymatic covalent crosslinks [13,14,19,37]. Together, fibril bundles create parallel ECM fiber alignment, and these fibers aggregate to form the ACL. Any disruption in this hierarchical structure, such as through mechanical overload or biochemical degradation, may compromise ACL ECM strength and increase injury risk [33]. Structural ECM characteristics are surrogates for ACL biomechanical properties and load distribution complexity. Both collagen crosslinking and total collagen content are key determinants of ACL biomechanical properties. By replacing damaged fibers, precise, slow collagen turnover rates help maintain ACL ECM structural integrity [38]. However, crosslink formation, rather than total collagen content, signifies ECM maturation level [39]. The tensile strength of collagen relates to its elongated, rod-like tropocollagen molecule composition assembled into a highly organized, repeating structure. The ACL is composed of several proteins, including collagen, elastin, and proteoglycans that are sensitive to oxidative modifications that can destabilize ECM structures. The oxidative stress caused by ROS accumulation leads to the genetic fibroblast dysfunction that impairs protein synthesis and increases protein degradation [34].

Although we do not currently know the extent to which an ACL that is damaged from accumulated microtrauma can repair itself [11,12], resting a mechanically fatigued tendon has been shown to improve mid-substance ECM remodeling [40]. Using microscopic and spectroscopic techniques, Chen et al. [11] observed that sudden ACL mechanical fatigue failure from accumulated microtrauma at its femoral enthesis resembled multiscalar injury damage that might have healed had it received sufficient repair time [11,12]. Interestingly, the ACL femoral enthesis insertion [11] is also the primary ACL vascularity and nerve innervation site [41]. In summary, impaired neurovascular function is associated with ECM collagen damage from accumulated microtrauma and compromised healing in the presence of insufficient active rest and recovery. The SORT evidence levels for this theme were 2 [33] and 3 [11,12,13,14,19,34,37].

### 3.3. The ACL Differs from Other Ligaments

Compared to the medial collateral ligament (MCL), the ACL has poor reparative capacity and is less responsive to growth factors [42,43]. The ACL also has less total collagen content than the posterior cruciate ligament (PCL), MCL, and lateral collateral ligament (LCL) [44], with less mature (suggesting greater turnover), more elastic collagen fibrils [45] passing through nonmineralized and mineralized fibrocartilage zones before attaching to bone. These fibrocartilaginous entheses consist primarily of type II collagen absorbing soft tissue–hard tissue interface loading stress [45]. The fact that a completely ruptured ACL has greater messenger ribonucleic acid (mRNA) for type I and III collagen production, and greater bigylcan concentrations than a healthy ACL, suggests that it actively attempts to heal, but without a lesion bridging scaffold, it fails [46]. Different knee ligaments also possess diverse biomechanical tensile strength properties. The patellar ligament has high tensile strength properties and the MCL and LCL have medium tensile strength properties, while the ACL and PCL tensile strength properties are comparatively lower [47,48]. In providing external reinforcement to the joint capsule, extra-capsular knee ligaments (MCL; LCL) are stiffer and stronger than intracapsular ligaments (ACL; PCL) [49]. In summary, in addition to poorer healing potential, the ACL has different histology and weaker biomechanical properties than other knee ligaments. The SORT evidence level for this theme was 3 [42,43,44,45,46,47,48,49].

### 3.4. Collagen, the ECM, and ACL Mechanobiology

The ACL is relatively hypocellular with a collagen-rich ECM and variable glycosaminoglycans (GAGs) concentrations [50]. Two-thirds of its total weight consists of interstitial fluid influencing its viscoelastic behavior [51]. Approximately 65–80% of ACL dry weight is composed of type I collagen, with the remainder consisting of proteoglycans, glycolipids, fibroblasts, and elastin [45,46,52,53]. Fibroblasts create collagen, and myofibroblasts are a subset of fibroblasts with alpha-Smooth Muscle Actin (α-SMA) cytoplasmic filaments that possess the capacity to contract and tension the ECM [46,54]. Higher myofibroblast concentrations are present at ACL injury sites [46,54]. Fibroblast mechanical loading influences ACL ECM alignment and orientation [55,56].

**Figure 1:**
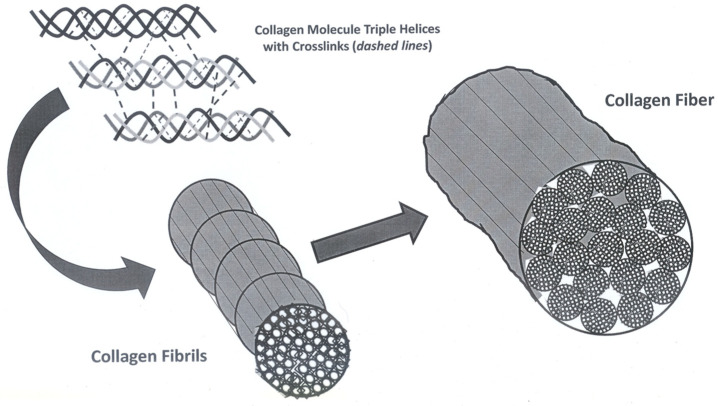
The hierarchy from collagen molecule to anterior cruciate ligament fascicle.

Within parallel collagen fibril bundles, fibroblasts form collagen fibers with most aligned in the direction of ACL tensile loads (Figure 1). Progressive collagen helix disruption reduces ACL tensile strength prompting abnormal, less organized fibril development. Following injury, fibroblasts upregulate ECM protein synthesis to repair damaged tissue [55], simultaneously producing enzymes that degrade the poorly organized ECM to synthesize a stronger, more functionally aligned and oriented tissue [55].

The ACL tends to follow the “use it” or “lose it” tissue integrity paradigm with positive ECM adaptations with increased loading, while loading below a minimal threshold weakens it [5,57]. For the ACL to recover from accumulated microtrauma, there must be a dynamic interplay between different cells, mediators (cytokines, growth factors, and protein cascades), and host physiology. Within a safe “physiologic window”, regular mechanical stimuli and high peripheral ECM collagen turnover are essential to ACL health. However, consistently decreased or excessively increased loading rates and frequencies can weaken the ECM [18].

Developing an improved understanding of how fibroblasts convert mechanical signals into the biochemical signals that modulate the gene expression that upregulates collagen synthesis is important to improve ACL ECM healing, better restore homeostasis, and prevent mechanical fatigue-related ACL injury [58,59,60]. In summary, ACL ECM biomechanical function is directly related to collagen fibril health. The SORT evidence level for this theme was 3 [5,18,45,46,50,51,52,53,54,55,56,57,58,59,60].

### 3.5. Crimps and ACL ECM Stretch

Regular sinusoidal “crimps” located near the ACL ECM bony insertions enable safe collagen fibril elongation to dampen knee joint loading forces [13,14]. As tensile loads increase, uncrimping occurs [13,14,61] and crimps progressively disappear. The non-linear ACL stress–strain curve “toe region” corresponds to crimp straightening [56]. The 1–3% stretch allowance provided by crimps enable the healthy ACL to resist sudden, potentially injurious tensile strains. A larger crimp angle is representative of a healthy, elastic, more functionally viable ACL ECM [13,14].

The ACL of the young athlete undergoes continuous growth and remodeling with growth referring to increased ECM mass, while remodeling refers to ECM mechanical property changes associated with microstructural maturation [32]. After exercise training, ACL ECM collagen formation and disorganized tissue degradation increases immediately with net collagen synthesis 36–72 h post-exercise [48]. Balanced post-exercise ACL growth and remodeling help reestablish natural stress–strain response homeostasis [32]. Mechanical stretch increases collagen and elastin degradation rates [32]. A 12% mechanical stretch increases lysyl oxidase (LOX) expression in both ACL and MCL fibroblasts, but to a greater extent in the MCL, contributing to its superior healing capacity [61,62]. Uniaxial cyclic ACL stretch (10 cycles/min, 10% length) increases collagen synthesis by increasing mRNA expression of type I and type III collagen [55]. In contrast, overstimulation or prolonged, excessive stress increases matrix metalloproteinase (MMP) activation, causing excessive degradation and reducing ACL ECM strength. For ACL growth and remodeling to maintain homeostatic equilibrium, collagen and elastin degradation and deposition rates must be equivalent [32]. When a target stretch level is met, fibroblasts deposit more collagen onto fibrils [32]. Collagen deposition increases the crimp fiber radius, while degradation decreases it [32]. In summary, crimp patterns in the femoral and tibial insertion regions of a healthy ACL ECM is essential to preserving its biomechanical properties during sudden loading. The SORT evidence level for this theme was 3 [13,14,30,48,55,56,61,62].

### 3.6. Crosslinks Improve ECM Mechanical Properties

**Figure 2:**
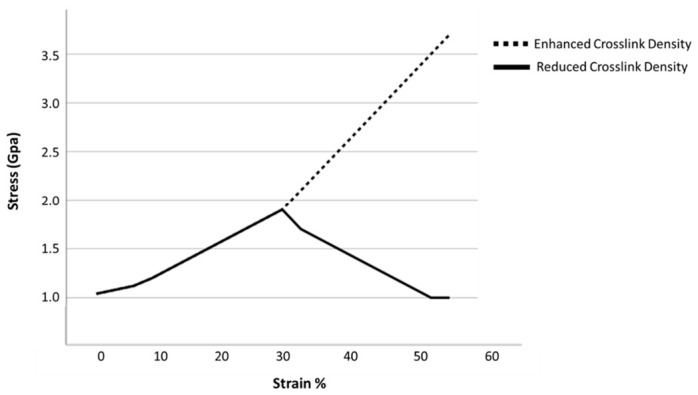
Stress–strain curve depiction of the stiffness and mechanical strength difference between an ECM with high, mature collagen fiber crosslink density and one with reduced density with more immature collagen fiber crosslinks. Original drawing based on reference [51].

The greater the ACL collagen network crosslink volume, the more it can synergistically deform, taking greater advantage of tropocollagen molecule mechanical properties. Fewer crosslinks make collagen fibers mechanically weaker and more friable [63]. A healthy crosslinked collagen fibril typically undergoes a three-phase loading behavior, allowing for greater deformation and energy dissipation prior to failure: 1. initial elastic deformation from collagen molecule uncoiling; 2. linear loading with intermolecular sliding; and 3. a second, stiffer elastic loading response associated with tropocollagen molecule “backbone” stretching until fibril rupture [64]. The increased stiffness observed during the second and third loading responses is determined by intermolecular divalent and trivalent crosslink network maturity, density, and orientation. Greater crosslink stiffness and strength enables a third deformation response. Stronger, mature crosslinks enable collagen molecules to deform beyond complete triple-helix uncoiling to achieve molecule backbone stretching [64] (Figure 2). Although total collagen content or density governs ultimate load mechanics, the density of mature crosslinks relates more to physiological ACL failure strain values [64]. Reducing the crosslink volume from 80% to 40% decreases the ultimate tensile strength and stiffness by 45% and 73%, respectively [64]. In summary, having sufficient active rest and recovery intervals between intense training sessions or competitions may improve collagen crosslink fiber density and maturity. The SORT evidence level for this theme was 3 [63,64].

### 3.7. The Delicate Collagen Synthesis and Degradation Balance

Following injury, an imbalance between ACL anabolic and catabolic processes contributes to knee microenvironment inflammatory changes [2]. Over time, the inflammatory synovial fluid milieu, coupled with altered metabolism contributes to post-traumatic knee OA, ultimately culminating in clinical signs of pain, inflammation, swelling, and functionally altered gait [2]. The Mitogen-Activated Protein Kinase/Extracellular Signal-Regulated Kinase pathway is an intracellular signaling cascade that translates mechanical signals from cell surface receptors to ACL cell nuclei, regulating cell proliferation, differentiation, survival, and collagen production by activating transcription factors that alter gene expression [33,65,66]. After collagen biosynthesis and triple-helix formation, copper-dependent LOX initiates covalent crosslink formation between ECM collagen fibrils, thus increasing ACL stiffness and strength, including resistance to matrix metalloproteinase (MMP) [67].

**Figure 3:**
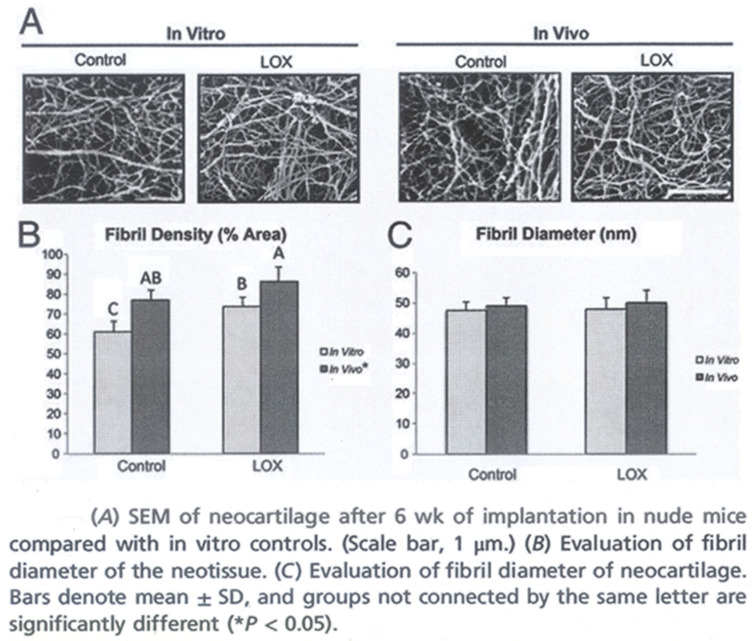
Exercise training and LOXL2 tend to increase collagen fiber crosslink density (**A**,**B**) more than diameter or total volume (**A**,**C**). Reprinted with permission from the Proceedings of the National Academy of Sciences of the United States of America [53].

Continuous collagen remodeling requires balanced LOX and MMP enzymatic activation. Fibroblast-produced LOX is the key ECM enzyme that catalyzes mature collagen crosslink development [25] (Figure 3). There are two general collagen crosslink types: LOX-controlled crosslinks (enzymatic crosslinks) and glycation or oxidation-induced advanced glycation end product (AGE) crosslinks [68]. Enzyme-dependent crosslinks strengthen collagen fibers and serve as a cell differentiation scaffold. As they mature, crosslinks form even stronger trivalent crosslinks, improving ECM mechanical strength [37]. A larger collagen fibril diameter and greater crosslink density indicate greater ECM tissue integrity. Crosslinks at collagen fibril surfaces increase ECM mechanical stiffness and load resistance [37,69]. Inhibited LOX activation reduces collagen crosslinking enough to reduce ECM tensile stiffness without altering total collagen or GAG content or macroscalar organization [70].

Through biochemical signals, transforming growth factor-beta (TGF-β) is a multi-functional cytokine that upregulates all LOX family members to facilitate ACL healing [25], crosslink formation, and ECM stabilization [71,72]. The stronger relationships that exist between ECM stiffness and mature enzymatic crosslink formation in an injured ACL compared to a healthy ACL support greater LOX-mediated crosslink importance during the healing and remodeling that occur following ACL degeneration and injury [69,73]. In the near future, advanced clinical imaging methods may be able to confirm high enzymatic crosslink ACL ECM densities and maturation levels, noninvasively confirming sufficient mechanical integrity for safer rehabilitation program advancement or return to sport participation [69]. LOX regulates ECM crosslink formation and modulates TGF-β and Epidermal Growth Factor receptor (EGFR) pathways. Exogenous TGF-β1 increases ACL collagen and fibronectin synthesis [74], serving an important ACL cell–ECM interaction role [75]. In homeostatic conditions, equilibrium exists between ECM synthesis and degradation [67] with balanced LOX and MMP expression being essential to healthy tissue remodeling [76].

Zinc-dependent MMP proteolytic enzymes are involved in ECM remodeling and repair through the proteolysis of collagens, elastin, and glycoproteins [55,67]. In addition to enzymatic activity, synovial fibroblasts further regulate ECM organization and protein synthesis by secreting growth factors [55]. Under normal conditions, MMP activity is tightly regulated to maintain tissue homeostasis. However, excessive mechanical ACL stress from increased anterior tibial translation can upregulate MMP expression, leading to increased collagen degradation and ACL ECM weakening [33]. Compared to fibroblasts in an injured MCL, following injury ACL fibroblasts display greater LOX expression decreases and MMP expression increases [62,67,77].

Poor injured ACL healing and remodeling has been linked to imbalanced synovial fibroblast MMP and LOX production [77,78]. Inflammatory cytokines also increase ACL fibroblast MMP expression or activation, impairing injured ACL healing [25,79,80]. Excessively high MMP levels and/or low LOX levels can also damage a healthy ACL, delaying its natural repair [77,81,82]. The inhibitory effects of Tumor Necrosis Factor-alpha (TNF-α) and Interleukin-1 beta (IL-1β) in synovial fluid may further reduce ECM crosslink volume with decreasing LOX concentrations weakening mechanical properties, and increasing MMP degradation susceptibility [77]. Greater collagen molecule crosslink density increases ECM resistance to collagenase mediated degradation by 2–3 times [66]. Inflammatory cytokines like TNF-α and IL-1β stimulate synovial fibroblasts to upregulate MMP-1, -2, and -3 activation to degrade injured ACL ECM collagen. Simultaneously, LOX expression is inhibited, severely limiting ACL structural repair capacity. A combination of ACL stretch-induced mechanical injury and inflammatory cytokine accumulation further suppresses LOX and amplifies MMP production. This “catabolic phenotype” imbalances the knee joint cavity synovial fluid environment to favor ACL tissue breakdown over regeneration [77]. Collagen turnover represents a delicate balance between synthesis and degradation by proteases such as collagenase MMPs [83]. Altered biosynthesis and degradative remodeling components impede ACL healing [77]. The combination of TNF-α and IL-1β downregulates LOX expression, decreasing synovial fluid LOX concentrations and thereby reducing ECM mature crosslink volume; this also increases its MMP degradation susceptibility, weakening it mechanically [77,84]. Exogenous TGF-β1 increases ACL fibroblast collagen and fibronectin synthesis and upregulates LOX activation [74,75]. In canine studies, the only biochemical difference between breeds with high or low ACL injury risk was that breeds with higher ACL rupture risk had higher degradative MMP2 levels with greater collagen turnover and greater ECM mature crosslink reduction [71]. In summary, subtle ACL biochemical changes may have a strong impact on the collagen anabolic–catabolic imbalances that lead to spontaneous mechanical fatigue-related ACL rupture. The SORT evidence levels for this theme were 2 [33] and 3 [2,25,37,53,55,62,65,66,67,68,69,70,71,72,73,74,75,76,77,78,79,80,81,82,83,84].

### 3.8. Exercise Training and the ACL

**Figure 4:**
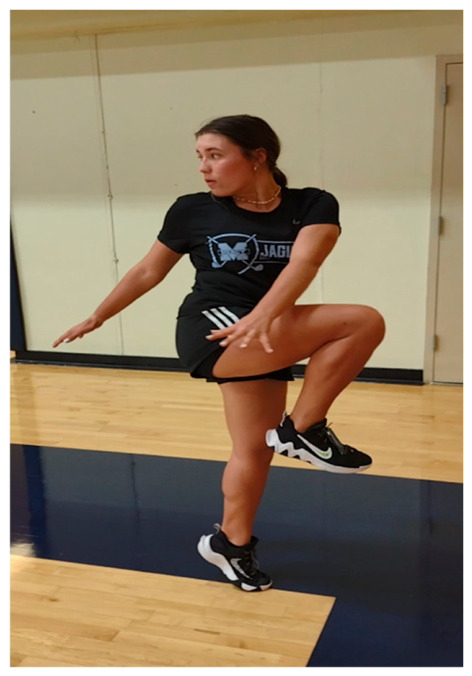
Active recovery, “form run simulation”. Since this athlete performs exaggerated functional movements without leaving the ground, this represents a low-impact, low-velocity movement task with high potential neuromuscular control, balance, and core–lower extremity coordination benefits with low injury risk. Load, pace, directionality, duration, feedback, and cues can be manipulated to better facilitate sport- and position-specific aerobic or anaerobic conditioning responses while concurrently limiting excessive knee joint loads.

In agreement with the “causal histogenesis theory” which states that tissue structure resembles the stresses to which it has been exposed [85], following immobilization or inactivity ACL mechanical properties deteriorate, collagen fibril size decreases, and crosslink density is reduced [36]. In response to exercise, ACL collagen synthesis increases, with men having higher inducible and baseline collagen turnover rates than women [47]. Within a physiologically safe “loading and unloading” window, regular exercise training facilitates stronger, more mature collagen network formation with increased crosslink density and maturity, better preserving ACL health and improving mechanical properties (Figure 4).

Among female basketball players (mean age = 19.9 years), Sakai et al. [86] reported a 2 mm (50%) physiologic anterior knee laxity increase after a 60 min warm-up and 150 min “game style” basketball practice. This laxity change decreased 90 min after basketball practice but remained elevated compared to pre-exercise values. By 5 h post-practice, laxity returned to pre-exercise values [86]. After a 40-week running program, male rabbit ACL strength increased [87]. After 8 weeks of motorized treadmill endurance exercise, male rat ACL strength increased [86]. Larsen et al. [88], however, reported that male rat ACL strength did not increase after a 6-week swimming program [88], and Sakuma et al. [89] reported that female rat ACL strength and stiffness was greater in rats that had not performed 4 weeks of daily treadmill running compared to those that did. They attributed these differences to the use of bipedal, not quadrupedal, rats and the use of higher-velocity tensile strength test loading rates more representative of impact loads. Previous studies reporting higher ACL exercise-induced failure loads and stiffness had relied on slower, non-physiologic loading rates [87,88,90]. Sakuma et al. [89] also reported greater ACL viscosity in the exercise group than in the non-exercise group, suggesting more brittle tissue properties [89]. Masujima et al. [91] studied the ACL stumps of female basketball athletes following acute ruptures. Light microscopy identified collagen fiber microtears, dilated ground substance, microcystic degeneration, and myxloid degeneration. Electron microscopy identified decreased amorphous ground substance and intracellular organelle destruction. These histological findings suggested that many ACL ruptures appeared to be associated with mechanical fatigue failure from continuously enforced exercise training [91,92]. Although a single low-to-moderate intensity exercise session likely has minimal effect on total collagen volume, prolonged or higher-intensity exercise training increases type I collagen turnover and net formation [93]. Acute exercise increases collagen catabolism, and 3–4 days post-exercise, type I collagen formation is elevated [91]. Signaling pathways convert ECM mechanical signals to gene expression and collagen synthesis [93].

Although regular physical activity generally benefits health, elevated blood and muscle ROS production biomarkers suggest that prolonged, high-intensity exercise may increase acute oxidative ECM damage [94]. In general, high-intensity, prolonged aerobic exercise (i.e., 65–75% VO2 max) results in greater ROS production than low-intensity (i.e., <40% VO2 max), short-duration exercise. Moreover, increased muscle temperature also results in higher ROS levels during contractions [95]. Although exercise levels that induce moderate ROS increases serve an important skeletal muscle adaptation role, excessive ROS production creates muscle damage that lessens physiological benefits [94]. Both short-term (5 consecutive days) and long-term (12 weeks) endurance exercise training increase antioxidant enzyme activity in trained muscles, reducing oxidative stress from an acute exercise bout [96]. Whether ROS production is damaging or beneficial depends on the level that occurs during exercise and the capacity of the cellular antioxidant system to protect ACL cells from oxidant damage [94]. A biphasic biological dose–response curve (hormesis) suggests that transient stressor elevation benefits ACL cellular effects, whereas a chronic or high dose leads to cell damage [94]. To sustain exercise intensity over repeated long-duration training sessions, nutrient-rich blood must be delivered to working tissues and ROS influence on mechanical tissue fatigue must be mitigated [94].

Since metabolic ACL health is vital to post-injury recovery, and exercise creates variable ROS levels, the rehabilitation exercises a patient performs after ACL injury should be monitored for ROS production [7,34]. Improved understanding of risk factors such as familial genetic predisposition and molecular pathways that interact with reduction-oxidation reaction molecules may provide novel primary ACL injury prevention and management insights [34].

Two groups of healthy young men were studied over 72 h following one hour of an acute non-damaging one-legged kicking exercise at 67% of the maximum workload [97]. There was a rapid increase in collagen synthesis after strenuous exercise in the human tendon and muscle peaking at 24 h [97]. In a murine model, protein synthesis increased 24 h after a single aerobic (animal treadmill) or resistance training (weighted ladder climbing) exercise bout, suggesting that exercise mode and the environment in which it is performed each influenced protein synthesis [98]. Four weeks of training upregulated Insulin-like Growth Factor 1 (IGF-1) levels and protein synthesis and reduced protein degradation and cell apoptosis [98]. Blood serum from human subjects after acute high-intensity resistance training (five leg press sets with 1 min between set rest intervals and three knee extension and hamstring curl supersets) applied to ligaments that were engineered from ACL cells increased collagen content, and maximal ligament tensile strength more than ligaments treated with serum from the same subjects at rest, with ICF-1 levels remaining unchanged [99]. Net collagen synthesis occurs 1–3 days post-exercise training [32,77] and is more influenced by increased amino acid, ascorbate (vitamin C), prostaglandin E2, and other hormone concentrations than solely by ICF-1 elevations [99]. In summary, although exercise is essential to ACL injury prevention and post-injury recovery, exercise prescription requires careful consideration, since intensity, duration, frequency, and total volume may contribute to homeostasis dysregulation [32]. The SORT evidence level for this theme was 3 [7,32,34,36,47,85,86,87,88,89,90,91,92,93,94,95,96,97,98].

### 3.9. Can Nutraceuticals Help Restore ACL Anabolic–Catabolic Balance?

Although high-intensity exercise training induces whole-body hormonal responses, how this biologically influences ACL healing and remodeling is unclear [99]. With enhanced bioavailability and targeted delivery, nano-nutraceuticals may support ECM growth and remodeling after training-induced ACL microtrauma [100]. Vitamin C, D, and E as well as certain polyphenols and terpenoids from fruits, vegetables, and herbs are potent antioxidants, antimicrobials, and anti-inflammatory agents that may help offset the harmful effects of ROS by creating a post-ACL microtrauma environment that is more conducive to tissue healing [33,34,101]. In summary, collagen supplementation may increase the amino acid concentrations needed for ECM repair, thereby helping restore anabolic–catabolic homeostasis [33]. The SORT evidence levels for this theme were 2 [33,101] and 3 [32,44,77,98,100].

### 3.10. Training Induced ACL Hypoxia

Sudden connective tissue rupture without clinical signs or symptoms such as pain or swelling most often occur at tissue degeneration sites with decreased vascularity from activity level and aging [102]. As in chronic tendinosus, decreased arterial blood flow and prolonged local hypoxia may disrupt the normal, low-level biochemical processes that maintain ECM repair, and supply energy to the AC [14,102]. Dietary habits that include high pro-inflammatory foods, or that lack essential collagen synthesis or other tissue repair nutrients, are key tissue degeneration and rupture factors. Mechanical stress from the ACL rubbing against the femoral intercondylar notch or roof, intercondylar tibial tubercles, or tibial plateau may also increase ROS accumulation, contributing to oxidative cellular component damage [33]. Chronic overuse tendon injuries arise from accumulated damage over repetitive loading cycles [103]. Oxidative stress in the ACL may impair fibroblast function, further promoting ECM degradation [33]. Although these underlying mechanism(s) are not well understood, they are likely related to MMP level increases and MMP inhibitor downregulation leading to greater ECM degeneration [103]. Chronic hypoxia may create the oxidative stress that leads to poor ACL healing. To prevent ECM degradation from oxidative stress and improve homeostasis, hypoxia pre-conditioned stem cell treatment has been reported to help regulate critical cellular signaling mechanisms that facilitate low oxygen level adaptations in combination with antioxidant or pharmacological agent ingestion [103]. Since weaker ECM properties in an otherwise healthy ACL are usually related to reduced mature crosslink density [51,104], it is important that sport training and athlete treatments support neo-crosslink development and maturation. Much needs to be learned about the optimal mode(s), timing, and frequency of therapeutic interventions for this purpose. Tendon and ligament ECM strength and maturation can be increased by Hypoxia-Inducible Factor-1 (HIF-1) regulated pathway stimulation, LOX gene expression, and sequential crosslink formation [53]. As hypoxia-induced LOX levels increase, crosslink density and ACL tensile properties also increase, reducing MMP-induced ECM degradation susceptibility and improving mechanical properties [53]. In summary, although intermittent hypoxia may enhance ACL ECM function, prolonged hypoxia may have a detrimental effect on ACL biomechanical characteristics. The SORT evidence levels for this theme were 2 [33] and 3 [14,51,53,102,103,104].

### 3.11. Estrogen and the Female Athlete

As estrogen levels increase, so does ACL laxity, with reduced ECM mechanical stiffness increasing ACL injury risk during pivoting directional change movements or single leg jump landings [105]. Whether from slower collagen volume increases or comparatively quicker crosslink formation, most mechanical and morphological ACL changes occur at the ECM periphery, with central ACL collagen fibers remaining the same from 17 years of age until death [106]. Increased estrogen concentrations do not negatively affect mechanical properties via altered collagen production [107] but rather through the decreased LOX activity that precedes decreased tissue stiffness [107]. After simulated exercise training, LOX mRNA concentrations increase in engineered ACL; however, when estrogen concentrations rise during the menstrual cycle follicular phase 3–4 days prior to ovulation, LOX activity and mechanical stiffness decrease, suggesting an elevated ACL rupture risk from decreased crosslink formation [107]. Physiologically elevated estrogen levels over a 48 h period decreases LOX activity 77% [107]. At a mechanistic level, molecular ACL regulatory functional processes are poorly understood [107]; however, these processes likely contribute to the increased non-contact ACL injury risk in athletically active females. In summary, monthly increased estrogen concentrations may adversely effect ACL ECM collagen crosslink density and maturity, negatively impacting its biomechanical properties. The SORT evidence levels for this theme were 2 [105,106] and 3 [107].

### 3.12. Counting Pitches or Counting Collagen Fiber Ruptures

Many young athletes with ligament or tendon overuse injuries regularly participate in high-intensity, frequency, duration, or total volume training over extended time periods. Counting the number of pitches thrown in a game has helped decrease youth baseball shoulder and elbow injuries. Other factors, however, such as high pitch velocity, high breaking ball pitch volume, faulty pitching mechanics, mechanically fatigued “at risk” tissues, poor nutrition, and limited active rest and recovery time between throwing sessions may also contribute to injury onset [108]. When a young baseball player pitches under “event fatigue” (too many pitches/game); “seasonal fatigue” (too many total pitches/season); or “year-round fatigue” (too many pitches/year), they have a 36 times greater chance of sustaining a throwing shoulder or elbow injury [109]. The most common cause of youth baseball upper limb injury is believed to be the year-round play that creates accumulated microtrauma at mechanically fatigued, repetitively stressed collagen dense joint connective tissues [109]. Rotator cuff tendons, elbow ligaments, and the ACL each represent vascularly challenged collagenous tissues that are frequently exposed to high-intensity, highly repetitive sports movement loads [110]. Factors such as early sport specialization, high training intensity, frequency and total volume, excessive focus on winning, prior injury history, familial genetic generalized laxity and family ACL injury predispositions, sex influences, anatomic variations, physiological fatigue, poor neuromuscular control, psychosocial stresses, extended “sports seasons”, and limited active rest and recovery intervals may each impede injury prevention strategies [100]. Conceivably, the active rest and recovery philosophy implemented in youth baseball may also help reduce sudden non-contact mechanical fatigue ACL ruptures in other youth sports. The SORT evidence level for this theme was 3 [100,108,109,110].

### 3.13. Restoring a Positive Anabolic–Catabolic Collagen Balance

Therapeutic modalities that promote tissue regeneration generally do so by improving cell growth, vascularity, nutrient delivery, metabolic waste removal, inflammatory response reduction, and cellular repair mechanism acceleration [33]. In any strategy to improve ACL ECM collagen balance, it is important to address all healing dimensions, including all regenerative processes from cellular signaling and ECM remodeling to the mechanical loading environment [33]. In the near future, clinical use of innovative ultrasound stress elastography and magnetic resonance imaging techniques may enable detailed microstructural ACL ECM histological and stiffness change assessments post-injury and during healing [33,111,112]. By combining insights from macroscale biomechanics with molecular-level studies, more effective ACL ECM strengthening strategies can be developed [33].

To more accurately predict sudden ACL mechanical fatigue rupture risk, biomarkers may help identify accumulated microtrauma ACL disease progression or resolution [2]. More comprehensive patient outcome-oriented clinical research studies are needed that evaluate the interplay between biological, mechanical, structural, and psycho-behavioral factors and ACL disease [2]. In summary, since accumulated microtrauma from overuse likely tips the ACL ECM anabolic–catabolic balance towards increased catabolism, monitoring active rest and recovery intervals may help facilitate homeostasis restoration. The SORT evidence levels for this theme were 2 [33,112] and 3 [2].

**Table 1:** Theme source evidence level SORT grade criteria [21]: High = high-quality systematic reviews of level 1 studies, randomized controlled trials, overall high quality, consistent patient-oriented outcome evidence; Moderate = lower quality, less patient-oriented outcome evidence; Low = pre-clinical basic science, animal models, consensus statements or guidelines, conceptual papers, theoretical models, or expert opinions, case series, simulation or mechanistic studies, observational studies, narrative or comprehensive reviews, and book chapters, with a more disease-oriented focus. Ref. # = reference number.

Theme	SORT Evidence Level [Ref. #]	Study Types [Ref. #]	Evidence Quality
A Hostile Environment, ACL Strain, and Poor Nutrient Delivery	2 [18,28,29,33]; 3 [2,6,7,16,17,23,24,25,26,27,30,31,32,34,35,36]	Prospective randomized [28]; case-control [18,29]; systematic review [33]; narrative or comprehensive review [2,6,23,27,30,34,35]; conceptual paper, theoretical model, or expert opinion [7,16,32,36]; animal model [17,26]; human basic science [24,25]; book chapter [31]	High = 0 Moderate = 4 Low = 16
Accumulative ACL Microtrauma and Mechanical Failure	2 [33]; 3 [11,12,13,14,19,20,34,37,38,39,40,41]	Systematic review [33]; cadaveric [11,20]; narrative or comprehensive review [12,34,40,41]; case-control [13]; histological [14]; molecular simulation [19]; human basic science [37,38]; biomaterials [39]	High = 0 Moderate = 1 Low = 12
The ACL Differs From Other Ligaments	3 [42,43,44,45,46,47,48,49]	Animal model [42,45,49]; human basic science [43,47]; cadaveric [44,46]; narrative or comprehensive review [48]	High = 0 Moderate = 0 Low = 8
Collagen, the ECM, and ACL Mechanobiology	3 [5,18,45,46,50,51,52,53,54,55,56,57,58,59,60]	Case-control [18]; animal model [45,53,54,56,57]; cadaveric [46,51]; narrative or comprehensive review [50,52,55,58,59,60]	High = 0 Moderate = 0 Low = 6
Crimps and ACL ECM Stretch	3 [13,14,32,48,55,56,61,62]	Case control [13]; histological [14]; conceptual paper, theoretical model, or expert opinion [32]; narrative or comprehensive review [48,55,56]; basic science mechanistic [61]; human basic science [62]	High = 0 Moderate = 0 Low = 8
Crosslinks Improve ECM Mechanical Properties	3 [63,64]	Animal study [63]; molecular computational model [64]	High = 0 Moderate = 0 Low = 2
The Delicate Collagen Synthesis and Degradation Balance	2 [33]; 3 [2,25,37,53,55,62,65,66,67,68,69,70,71,72,73,74,75,76,77,78,79,80,81,82,83,84]	Human basic science [25,37,62,66,67,74,75,76,77,78,79,80,83]; animal model [53,68,69,71,72,82]; systematic review [33]; narrative or comprehensive review [55,65,73]; case-control [70,81]; mathematical computational [84]; conceptual paper, theoretical model, or expert opinion [85]; cross-sectional [86]	High = 0 Moderate = 1 Low = 20
Exercise Training and the ACL	3 [7,32,34,36,47,85,86,87,88,89,90,91,92,93,94,95,96,97,98]	Conceptual paper, theoretical model, or expert opinion [7,32,36]; narrative or comprehensive review [34,93,94,95]; human basic science [47,91]; animal model [87,88,89,90,98]; observational [92]; systematic review [96]; cohort [97]	High = 0 Moderate = 0 Low = 19
Can Nutraceuticals Help Restore the Balance?	2 [33,101]; 3 [32,44,77,99,100]	Systematic review [33]; conceptual paper, theoretical model, or expert opinion [32]; cadaveric [44]; human basic science [77,99]; narrative or comprehensive review [100]	High = 0 Moderate = 2 Low = 5
Training Induced ACL Hypoxia	2 [33]; 3 [14,51,53,102,103,104]	Histological [14]; systematic review [33]; cadaveric [51]; animal model [53,104]; narrative or comprehensive review [102,103]	High = 0 Moderate = 1 Low = 6
Estrogen and the Female Athlete	2 [105,106]; 3 [107]	Cohort [105,106]; human basic science [107]	High = 0 Moderate = 2 Low = 1
Counting Pitches or Counting Collagen Fiber Ruptures	3 [100,108,109,110]	Narrative or comprehensive review [100,110]; conceptual paper, theoretical model, or expert opinion [108,109]	High = 0 Moderate = 0 Low = 4
Restoring A Positive Anabolic–Catabolic Collagen Balance	2 [33,112]; 3 [2]	Narrative or comprehensive review [2]; systematic review [33,112]; cadaveric study [111]	High = 0 Moderate = 2 Low = 2

## 4. Study Limitations

This narrative review thematically summarizes evidence supporting the importance of maintaining ACL ECM anabolic–catabolic balance in preventing spontaneous non-contact, mechanical fatigue-related ACL rupture. Contributing evidence, however, consisted solely of low and moderate SORT level sources. This included heterogenous reviews, cohort, case-control, case series, observational, pre-clinical basic science, animal model, conceptual papers, theoretical models, expert opinions, simulation or mechanistic studies, and book chapters, often with a more disease-oriented focus (Table 1). This foundational evidence sets the stage for developing higher-level patient outcome-oriented randomized controlled trial and prospective cohort studies. Studies such as these will help establish more prescriptive active rest and recovery and therapeutic intervention mode(s), timing, and dosage guidelines.

## 5. Conclusions

More prescriptive active rest and recovery intervals and neuromuscular control training may restore the anabolic–catabolic balance that increases mature crosslink density and improves ACL ECM strength. Confirmatory studies are needed to better establish therapeutic intervention mode(s), timing, dosage, and frequency optimization.

## Data Availability

No new data were created or analyzed in this study.

## Abbreviations

The following abbreviations are used in this manuscript:

ACL	Anterior cruciate ligament
BNIP3	BCL2/adenovirus E1B 19-kDa-interacting protein 3
ECM	Extracellular matrix
OA	Osteoarthritis
ROS	Reactive oxygen species
MCL	Medial collateral ligament
LCL	Lateral collateral ligament
PCL	Posterior cruciate ligament
mRNA	Messenger ribonucleic acid
GAGs	Glycosaminoglycans
α-SMA	alpha-Smooth Muscle Actin
LOX	Lysyl oxidase
MMP	Matrix metalloproteinases
PYR	Pyridinoline
AGEs	Advanced glycation end products
TGF-β	Transforming growth factor-beta
EGFR	Epidermal Growth Factor receptor
TNF-α	Tumor Necrosis Factor-alpha
IL-1β	Interleukin-1 beta
IGF-1	Insulin-like Growth Factor 1
VEGF	Vascular Endothelial Growth Factor
